# EVOLUTION OF THE BRAZILIAN JOURNAL OF CARDIOVASCULAR SURGERY

**DOI:** 10.21470/1678-9741-2022-0956

**Published:** 2022

**Authors:** Paulo Roberto B. Evora, Andreia C. Feitosa do Carmo

**Affiliations:** 1 Editor-in-Chief - BJCVS; 2 Faculdade de Medicina de Ribeirão Preto da Universidade de São Paulo (FMRP-USP), Ribeirão Preto, SP, Brazil; 3 Hospital São Paulo, Escola Paulista de Medicina da Universidade Federal de São Paulo (EPM-Unifesp), São Paulo, SP, Brazil

The *Brazilian Journal of Cardiovascular Surgery* (BJCVS) is the renowned journal of the Brazilian Society of Cardiovascular Surgery (SBCCV). BJCVS is a bimonthly, peer-reviewed clinical journal, published on a regular basis since 1986. BJCVS aims to report clinical developments and innovation in cardiovascular surgery as well as to seek professional improvement and updating in the area. It has a considerable impact on the practice of cardiovascular surgery and associated areas.

In an editorial published by the BJCVS^[1]^, we presented our views about the impact factor (1,312), provided by the Journal Citation Reports (JCR). However, we believe that readers of this journal should be aware that BJCVS is also indexed by Scopus, another global citation database. As JCR generates a list of journals indexed by Web of Science, SCImago annually generates a list of journals indexed by Scopus, which are sorted by their SCImago Journal Rank (SJR) for each of its subject categories. Hence, according to SCImago data, BJCVS is ranked as Q3 in three subject categories: “Cardiology and Cardiovascular Medicine”, “Medicine (miscellaneous)”, and “Surgery”.

**Figure 1:**
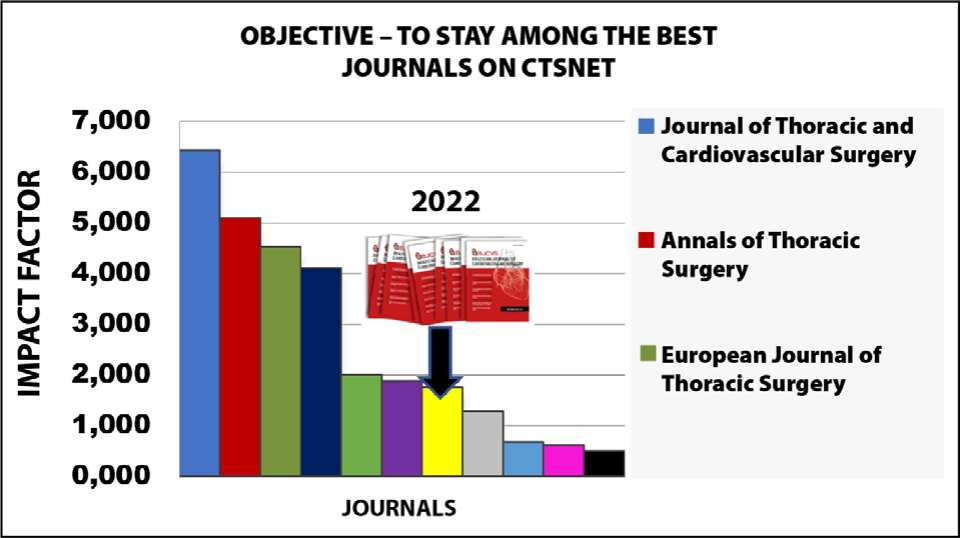
BJCVS position among the Cardiothoracic Surgery Network (CTSNET) journals.

**Figure 2:**
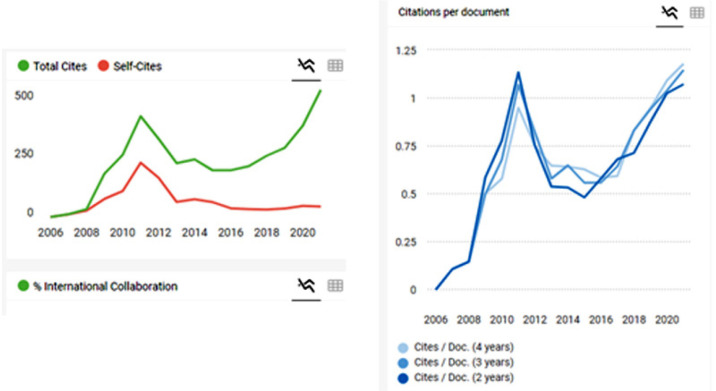
Metric numbers confirm the continuous increase. Left: Total documents and Total cites/self-citations; Right: Citations per document.

SJR is a portal with scientometric indicators of journals indexed in Scopus. All data have been provided by Scopus/Elsevier and SCImago has no authority over this data, which are property of Scopus/Elsevier. SCImago has a signed agreement that limits our performance to the generation of scientometric indicators derived from the metadata sent in the last update. Reflecting the increase in quality, the 2021 impact factor reinforced the trajectory of the BJCVS among the best-rated publications (Figure 1, Table 1). Other important metric numbers also confirm the continued increase: 1) Citations per document, 2) Total documents and 3) Total cites/self-citations (Figure 2).

It is mandatory to value the work and expertise of national and international authors for the production of quality articles that allow BJCVS to occupy, among the journals selected by CTSnet according to its impact factor, the 7th position in 2021 and the 8^th^ position in 2022 (Figure 1, Table 1).

**Table 1:** Cardiothoracic Surgery Network (CTSNET) journals.

CTSNET JOURNALS	IF 2021	IF 2022
1. Journal of Thoracic and Cardiovascular Surgery	5.220	6.439
2. Annals of Thoracic Surgery	4.330	5.102
3. European Journal of Thoracic Surgery	3.486	4.534
4. Annals of Cardiothoracic Surgery	4.101	4.110
5. Seminars in Thoracic and Cardiovascular Surgery	1.190	2.006
6. Journal of Cardiovascular Surgery	1.550	1.888
7. General Thoracic and Cardiovascular Surgery	1.088	1.756
8. Brazilian Journal of Cardiovascular Surgery	1.312	1.283
9. Heart Surgery Forum	0	0.676
10. Operative Techniques in Thoracic and Cardiovascular Surgery	0.619	0.620
11. Asian Cardiovascular and Thoracic Annals	0.49	0.510

We are grateful for the relevant and continuous support of the Brazilian Society of Cardiovascular Surgery, and the commitment and dedication of the Associate Editors and reviewers, responsible for the improved quality of the BJCVS.

Finally, why are we in debt to our contributors and readers? We are currently working far beyond our editorial capacity. We have over 80 ahead of print articles, and continue to receive 40 to 60 new submissions monthly. This situation makes our peer-review system increasingly selective, and we often reject excellent papers. Using some comments from our Associate editors, manuscripts are not accepted for publication for many reasons. Some may relate directly to scientific reasons; others may be more closely related to the perceived interests of our readers, priority for publication, and number of manuscripts awaiting publication. Often these decisions do not reflect the quality of the work being submitted. In view of this scenario, we believe that we should seek to remain among the best CTSNET journals. To improve academic importance, it will be necessary to set up a more effective structure.

In conclusion, the BJCVS remains on the right track^[1-3]^. However, the saga continues and everyone’s commitment is necessary to continue these advances.
